# Colorectal cancer incidence, survival analysis and predictions (Monastir, Tunisia: 2002–2030)

**DOI:** 10.1371/journal.pone.0339603

**Published:** 2026-01-13

**Authors:** Cyrine Bennasrallah, Amani Maatouk, Mariem Kacem, Wafa Dhouib, Hela Abroug, Manel Ben Fredj, Leila Safer, Sonia Zaied, Moncef Mokni, Ines Bouanène, Asma Belguith Sriha, Imen Zemni

**Affiliations:** 1 Department of Epidemiology and Preventive Medicine, Faculty of Medicine of Monastir, University Hospital Fattouma Bourguiba of Monastir, University of Monastir, Monastir, Tunisia; 2 Faculty of Medicine of Monastir, Technology and Medical Imaging Research Laboratory - LTIM - LR12ES06, University of Monastir, Monastir, Tunisia; 3 Hygiene Department, Faculty of Medicine of Monastir, University Hospital of Monastir, Monastir, Tunisia; 4 Department of Community and Preventive Medicine, Faculty of Medicine of Monastir, Regional Hospital of Ksar Hellal, Monastir, Tunisia; 5 Department of Gastroenterology, Faculty of Medicine of Monastir, University Hospital Fattouma Bourguiba of Monastir, Monastir, Tunisia; 6 Department of Oncology, Faculty of Medicine of Monastir, University Hospital Fattouma Bourguiba of Monastir, Monastir, Tunisia; 7 Cancer Registry of the Center of Tunisia, University Hospital Farhat Hached of Sousse, Sousse, Tunisia; Faculty of Medicine of Monastir, TUNISIA

## Abstract

**Background:**

Colorectal cancer (CRC) ranks as the third most common cancer and the second leading cause of cancer death in Tunisia. The aim of this study was to describe the incidence and mortality rates, to determine CRC trends, prediction and burden and to analyses the CRC survival outcomes.

**Methods:**

We conducted population-based cohort study.of all CRC cases diagnosed between 2002 and 2014 in the governorate of Monastir, with follow-up until 2022. Data were obtained from the Central Cancer Registry, the Hospital Morbidity and Mortality Registry, and national mortality databases, ensuring comprehensive case identification. Incidence, mortality, disability-adjusted life years (DALYs), Annual Percentage Change (APC), and survival were analyzed using standardized methods.

**Results:**

We identified 876 cases of CRC. The age-standardized incidence rate (ASIR) was 13.5/100,000 PY (95% CI: 9.8–17.2), with a significant upward trend (APC = +6.09% (95% CI: + 0.64; + 11.82).). The predicted ASIR for 2030 was 60.4 per 100,000 PY (95% CI: 52.0–68.2). The rectum being the most frequent site (32.9%), followed by the recto-sigmoid region (28.3%). The age-standardized mortality rate was 9.6/100,000 PY (95% CI: 6.5–12.7). The DALY/year/ 100,000 PY was 96.0 (86.2–105.8). Survival rate was 64% (95% CI: 62.3–66.2), at one year and 21.1% (95% CI: 19.3–22.9) at five years.

**Conclusion:**

CRC incidence and mortality in Monastir have increased significantly, with projections indicating a continued rise by 2030. Strengthening population-based screening and early detection could help reverse these trends and improve prognosis.

## Introduction

Colorectal cancer (CRC) accounts for 10% of all cancers and is the second leading cause of cancer death worldwide [1,2]. The incidence of colorectal cancer (CRC) varies significantly worldwide, with differences of at least 25-fold between countries [3]. The highest rates were observed in high-income countries (HICs). The incidence raised sharply in low- and middle-income countries (LMICs) [4]. By 2040, CRC is expected to reach 3.2 million new cases and 1.6 million deaths annually, [5], especially in LMICs [6]. While HICs are benefiting from declining mortality related to screening and innovative therapies, LMICs face an increasing burden with limited resources, highlighting the urgent to address global health disparities [7]. Over the past two decades, lifestyle shifts in LMICs (foods, physical activity, obesity) have mirrored patterns in HICs, amplifying CRC risk [8]. Modern lifestyle model, limited screening, and delayed diagnoses further extend disparities in LMICs [9]. In Tunisia, CRC represents a major public health concern, ranking as the third most common cancer and the second leading cause of cancer-related death [10]. According to GLOBOCAN 2022, the age-standardized incidence (ASIR) and mortality rates (ASMR) were 11.9 and 6 per 100,000, respectively [10]. To address this burden, a national CRC screening program based on the fecal immunochemical test (FIT) was launched in 2016, with colonoscopy recommended for positive cases. However, data on CRC incidence and trends remain limited. This study explores key epidemiological aspects of CRC in Tunisia, focusing on the Monastir region. It analyzes incidence trends from 2002 to 2014, evaluates survival outcomes up to 2022, and provides projections through 2030.

## Methods

### Study design

We conducted a population-based cohort study, tracking all CRC patients diagnosed between 2002 and 2014 in the governorate of Monastir (Tunisia), with follow-up until 2022.

### Setting

This study was conducted in the governorate of Monastir, considered a representative Tunisian governorate. The prevalence of obesity in the region was 28.11% (95%CI 27.42%−28.58%) [11], while the prevalence of eating disorders was markedly high, reaching 48% (95% CI: 43–52), particularly among women [12]. The national CRC screening program using the fecal immunochemical test (FIT) targets individuals at high risk of CRC. Patients with confirmed CRC following colonoscopy were subsequently managed by gastroenterologists and oncologists. The Central Cancer Registry systematically records all cancer cases from the central region of Tunisia, including those from the governorate of Monastir. In parallel, the Hospital Morbidity and Mortality Registry, managed by the Department of Preventive Medicine and Epidemiology, documents all hospital-based morbidity and mortality cases, including cancer cases, from the only university hospital center in the governorate. Between 2002 and 2014, all cancer cases in the region were exclusively managed within public health facilities. A collaborative framework between the Central Cancer Registry and the Hospital Morbidity and Mortality Registry was established to ensure exhaustive identification and documentation of cancer cases in Monastir. Furthermore, the National Authority for the Protection of Personal Data granted access to mortality data for the governorate of Monastir, contingent on strict adherence to confidentiality agreements.

### Participants

Inclusion Criteria: This study included all cases of CRC diagnosed in patients residing in the governorate of Monastir between 2002 and 2014, including those diagnosed and treated outside the region. Participant follow-up was ensured through linkage with the Monastir all-cause mortality database, spanning the years 2001–2022.

Exclusion Criteria: Benign and in situ tumors, tumor recurrences, and the metastatic progression were not included in the study.

### Variables

Tumors were classified using the ICD-10, with code C180 to C209 assigned to malignant neoplasms of the CRC. For analysis, the dataset included key variables such as date of birth, sexes, year of diagnosis, CRC topography and morphology, the status (deaths, survival) and the year of death. We classified colorectal cancer (CRC) into right- and left-sided tumors. Right-sided CRC included lesions of the ileocecal junction, ascending colon, and transverse colon, whereas left-sided CRC included tumors of the descending colon, sigmoid colon, and rectum [13].

### Data collection and mesurements

Data on CRC cases diagnosed between 2002 and 2014 were prospectively collected by merging the hospital morbidity registry data and the regional cancer registry of the Center. The team of the Department of Epidemiology and Preventive Medicine of Monastir verified the data conformity in décember, 2019.

In a second step, deaths among these cases and their corresponding dates were ascertained using multiple sources: the hospital death database and the Ministry of Social Affairs mortality database, which records all deaths in Monastir between 2001 and 2022. The study protocol was approved by the Ethics Committee of the Faculty of Medicine of Monastir in January 2022, and access to the mortality database was authorized in May 2022 following approval from the National Authority for the Protection of Personal Data. The mortality dataset comprised 60,280 deaths registered in the Monastir governorate between January 1, 2002, and January 26, 2023 (data accessed in February 2023). For unresolved cases, a name-by-name verification was performed in the municipal death registry, which provides a systematic record of all deaths in Tunisia (S1 Appendix).

The Crude Incidence Rate (CIR) and the Crude Mortality Rates (CMR) were calculated based on Tunisian National Institute of Statistics data and was expressed per 100,000 inhabitants [14]. The ASIR and Age-Standardized Mortality Rate (ASMR) per 100,000 person-years (PY) was calculated using the world standard population according to the World Health Organization statement of 2013 [15].

For the Disability-Adjusted Life Years (DALYs) calculation: Years of Life Lost (YLL) were calculated by multiplying the number of cancer-related deaths by the standard life expectancy at the age of death, using 79 years for women and 74.2 years for men as reference life expectancies at birth. Years Lived with Disability (YLD) were estimated by multiplying the number of prevalent cases by the disability weight of the mean of 0.238_0.209, given that cancer stage is not specified in our database [16].

### Statistical analysis

Data were verified and analyzed using IBM SPSS Statistics for Windows, Version 20.0. The chi-squared test for independent samples was used to compare incidence, mortality and survival rates. To test trends of the CIR we have calculated the Annual Percentage Change (APC) using Join point regression program. For the projections, we used SPSS with a grouped dataset: the first variable represented year (2002–2030), followed by grouped counts for all cases, by sex, and by age categories. We applied a Poisson log-linear regression within the generalized linear model (GLM) framework, with the observed counts as the dependent variable and year as a covariate, including main effects. Predicted counts with 95% confidence intervals were generated from the fitted model. Subsequently, Excel was used to calculate predicted ASIR. We recalculated the predicted ASIR using the formula


$${\text{ASIR}}_{t\;}={\text{ASIR}}_{baseline}\times {\left(\text{1}+APC\right)}^{\left(t-baseline\right)}$$

to verify the results obtained from the SPSS Poisson log-linear regression within the generalized linear model (GLM).

Survival curves were generated using the Kaplan–Meier method, using the log-rank test to assess differences in survival between various sub-groups. A p-value < 0.05 was considered statistically significant.

### Ethics approval and consent to participate

The study was conducted in accordance with recognized ethical standards. Ethical approval was obtained from the Ethics Committee of the Faculty of Medicine of Monastir, Tunisia (Reference Number: IORG0009738 N°101/OMB0990–0279). All participants provided written informed consent for the use of their health data for scientific research prior to inclusion in the study. To ensure confidentiality, all data were anonymized before analysis. The data supporting the findings of this study are publicly available on Zenodo at the following DOI: https://doi.org/10.5281/zenodo.17367754.

## Results

### Digestive tract neoplasms (2002–2014)

A total of 1,119 cases of digestive tract cancers were recorded, corresponding to a CIR of 11.8 per 100,000 inhabitants (95% CI: 8.3–15.2).The CRC represented 73.1% of all the digestive tract cancers. Stomach Cancers represented 21.8% of cases (n = 261) with a CIR of 3.50 per 100,000 inhabitants (95% CI: 1.6–5.37) (Table 1).

**Table 1:** Incidence of the digestive tract neoplasms, Monastir, (2002–2014).

Cancer type	N (%)	CIR (95% CI)*
All	1,199	16.08 (12.1-20.1)
Colorectal Cancer	876 (73.1)	12.72 (9.2-16.3)
Stomach Cancer	261 (21.8)	3.50 (1.6-5.37)
Esophageal Cancer	26 (2.2)	0.35 (0.0-0.94)
Intestinal Cancer	25 (2.1)	0.34 (0.0-0.91)
Anal Cancer	11 (0.9)	0.15 (0.0-0.53)

*: per 100000 inhabitants; CIR

### Colorectal cancer incidence (2002–2014)

During 12 years, 876 cases of CRC were recorded (67 cases per year) in Monastir. Sex ratio was 1.12 (p = 0.105). The median age at CRC diagnosis was 58 years (Q1-Q3: 45–70). It was 58 years (Q1-Q3: 47–71) in men and 57 (Q1-Q3: 43–69) in women (p = 0.239). Median age stable was stable during our study period (p = 0.149).

The CIR was 12.7/100,000. It was 66.9 per 100,000 in the elderly. ASIR was 13.5/100,000 PY (95% CI: 9.8–17.2). It was 14.9/100,000 (95% CI: 11.0–18.8) among males and 11.81/100,000 (95% CI: 8.4–15.2) in females (Table 2).

**Table 2:** Colorectal cancer incidence and mortality by age groups and gender, Monastir (2002−14; 2022).

CCR Morbidity	All			Males			Females		
	N (%)	CIR (95% CI)	ASIR (95% CI)	N (%)	CIR (95% CI)	ASIR (95% CI)	N (%)	CIR (95% CI)	ASIR (95% CI)
All	876 (100)	12.72 (9.2-16.3)	13.53 (9.8-17.20)	462 (52.7)	13.47 (9.8-17.1)	14.90 (11.0-18.8)	414 (47.3)	11.99 (8.5-15.5)	11.81 (8.4-15.2)
Age groups									
< 20	41(4.7)	1.72 (0.4-3.04)		22 (4.8)	1.79 (0.5-3.13)		19 (4.6)	1.65 (0.4-2.94)	
20-39	105 (12.2)	4.59 (2.4 −6.73)		42 (9.2)	3.80 (1.8-5.8)		63 (15.4)	5.32 (3.0-7.63)	
40-64	409 (47.3)	23.34 (18.5-28.17)		224 (49.2)	25.22 (20.2-30.3)		185 (45.2)	21.40 (16.8-26.02)	
≥ 65	309 (35.8)	66.93 (58.7- 75.11)		167 (36.7)	80.64 (71.7-89.6)		142 (34.7)	55.77 (48.3-63.24)	
**CCR Mortality**	**All**			**Males**			**Females**		
	**N (MR)**	**CMR (95% CI)**	**ASMR (95% CI)**	**N (MR)**	**CMR (95% CI)**	**ASMR (95% CI)**	**N (MR)**	**CMR (95% CI)**	**ASMR (95% CI)**
All	622 (71.0)	9.04 (6.0–12.0)	9.61 (6.5-12.71)	324 (70.1)	9.44 (6.4–12.5)	10.60 (7.3-13.86)	298(72.0)*	8.63 (5.7–11.6)	8.66 (5.7-11.60)
Age groups at death									
< 20	8 (19.5)	0.34 (0-0.93)		4 (17.6)	0.32 (0.0-0.09)		4 (23.1)	0.38 (0.0-1.0)	
20-39	43(40.9)	1.88 (0.5-3.25)		16 (37.0)	1.41 (0.2-2.59)		27 (43.6)	2.32 (0.8-3.84)	
40-64	266 (65.0)	15.20 (11.3-19.1)		150 (67.1)	16.92 (12.8-21.03)		116 (62.5)	13.37 (9.7-17.03)	
≥ 65	282 (91.3)	61.13 (53.3-68.9)		145 (86.6)	69.84 (61.5-78.2)		136 (95.9)	53.47 (46.2-60.8)	

CIR/ CMR: Crude Incidence Rate/ Crude Mortality Rate; ASIR/ ASMR: Age-Standardized Incidence Rate/ Age-Standardized Mortality Rate; 95% CI: 95% Confidence Interval.

#### Colorectal cancer incidence trends.

**Fig 1:**
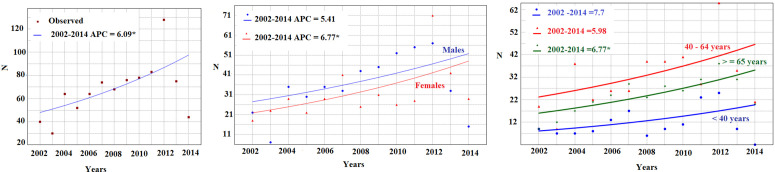
Trends in colorectal cancer incidence by sex and age group, 2002–2030. The overall trend showed a significant increase in colorectal cancer incidence. Stratified analyses indicated a significant upward trend among individuals aged 65 years and older and a notable increase among women.

The CIR of CRC exhibited a statistically significant upward trend, with an APC of +6.09% (95% CI: + 0.64; + 11.82). This rise was equal in both sexes (Fig 1). It was significantly high among elderly (APC = +6.77% (95% CI: + 2.09; + 11.67) (Table 3).

**Table 3:** Trends and prediction of colorectal cancer incidence (at 2030).

	Trends	Prediction 2025		Prediction 2030	
		CIR^$^ (95% CI)	ASIR^$^ (95% CI)	CIR^$^ (95% CI)	ASIR^$^ (95% CI)
All	+6.09% (+0.64; + 11.82).	27.72 (22.5-33.0)	40.00 (33.7-46.3)	35.91 (29.9-41.9)	60.41 (52.-68.2)
< 40	+7.7 (−1.7; + 18.1)	10.3 (7.1-13.5)		12.3 (8.8-15.8)	
40-64	+6.0 (−1.0; + 13.5)	54.09 (46.7-61.4)		71.21 (62.8-79.5)	
≥ 65	+6.8 (+2.1; + 11.7)*	181.94 (168.5-195.4)		252.11 (236.3-268.0)	
Gender					
Male	+5.4 (−1.7; + 13.0)	26.78 (17.7-40.3)	52.00 (44.8-59.2)	33.72 (19.8-57.4)	90.04 (80.6-99.5)
Female	+6.8 (+1.6; + 13.3)*	29.03 (18.9-44.8)	36.27 (30.2-42.3)	38.83 (22.0-67.9)	52.96 (45.7-60.2)

95% CI: 95% Confidence Interval; CIR: Crude Incidence Rate; ASIR: Age-Standardized Incidence Rate.

#### Colorectal incidence prediction to 2030.

By 2030, in the study region, the estimated number of new cases is projected to reach 206 per year (95% CI: 140–303). The predicted ASIR for 2030 was 60.4 per 100,000 PY (95% CI: 52.0–68.2) based on the Poisson log-linear regression, and 61.6 per 100,000 PY (95% CI: 44.2–77.6) when calculated using the standard APC formula. Among men, the projected ASIR is expected to reach 90.04/100,000 PY (95% CI: 80.6–99.5)in 2030 (Table 3).

### Colorectal cancer topography and morphology

**Fig 2:**
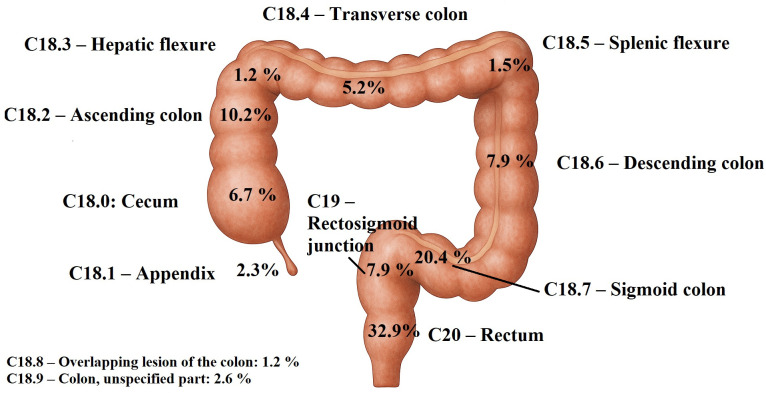
Distribution of colorectal cancer cases by anatomical site and histopathological type. Left-sided colorectal cancers accounted for the majority of cases with the rectum being the most frequent site (32.9%). Adenocarcinoma was the predominant histopathological type.

Left-sided colorectal cancers represented the majority of cases (70.6%), with the rectum being the most frequent site (32.9%), followed by the recto-sigmoid region (28.3%) (Fig 2). Adenocarcinoma constitutes the predominant histopathological type (96% of cases), of which 35% are of the intestinal subtype and 56% are unspecified.

### Colorectal cancer mortality (2002–2022)

During 20 years study, 622 cases of CRC dead (71%). Sex ratio was 1.12 (p = 0.219). The median age at CRC death was 69 years (Q1-Q3: 55–78), it was 68 years in males (Q1-Q3: 54–77) and 70 in females (Q1-Q3: 55–79) (p = 0.280). The CMR was 9.0/100,000. It was 9.4/100,000 in males and 8.6/100,000 in females. The majority of deaths occurred in the elderly (CIR: 61.1 per 100,000 inhabitants). ASMR was 9.6/100,000 PY (95% CI: 6.5–12.7). It was 10.6/100,000 (95% CI: 7.3–13.9) among males and 8.7/100,000 (95% CI: 5.7–11.6) among females (Table 2).

#### Disability-adjusted life years attributable to colorectal cancer.

The burden of DALYs due to CRC totals 6,609 DALYs, (96.0/ 100,000 PY) with 91.8% attributable to years of life lost (YLLs). By age group, the largest contribution to DALYs is observed in individuals aged 40–64 years (60% of the total; 3,966 DALYs) Table 4.

**Table 4:** Burden attributable to colorectal cancer by age group and sex (2002−14; 2022).

	DALY	
	Totals*	DALY/year/ 100,000 PY
All	6609 (100)	96.0 (86.2 −105.8)
< 20	404 (6.1)	17.0 (12.9-21.1)
20-39	1256 (19.0)	54.9 (47.5–62.3)
40-64	3966 (60.0)	226.3 (211.3–241.3)
≥ 65	983 (14.9)	212.9 (198.3–227.5)
Gender		
Male	3250 (49.2)	94.7 (85.0–104.5)
Female	3359 (50.8)	97.3 (87.5–1072)

*Cohort 2002–2014 followed to 2022; DALY: Disability-Adjusted Life Years

### Survival analysis of colorectal cancer

At the overall level, the one-year survival rate among all colorectal cancer patients was 64% (95% CI: 62.3–66.2), decreasing to 48.3% (95% CI: 46.3–50.0) at three years and 21.1% (95% CI: 19.3–22.9) at five years. Patients less than 40 years had the highest survival at one year (83.3%, 95% CI: 79.5–87.1), three years (74.0%, 95% CI: 69.5–78.5), and five years (59.7%, 95% CI: 53.8–65.6), whereas those ≥65 years had the lowest survival (one year: 57.1%, 95% CI: 54.0–60.2; three years: 39.0%, 95% CI: 35.9–42.1; five years: 4.9%, 95% CI: 3.5–6.3). Survival was similar between males and females, with five years survival of 22.2% (95% CI: 19.6–24.8) and 20.0% (95% CI: 17.3–22.7), respectively. Patients with cecal tumors (C18.0) showed the highest one year (66.6%, 95% CI: 65.4–67.8) and five years survival (24.2%, 95% CI: 22.9–25.5), while recto-sigmoid junction tumors (C19.9) had the lowest one year (45.0%, 95% CI: 33.9–56.1) and five years survival (6.0%, 95% CI: 1.8–10.4). Rectal cancers (C20.9) were associated with intermediate survival (one year: 62.5%, three years: 42.2%, five years: 16.5%) (Table 5).

**Table 5:** Cumulative survival proportion at the end of the 1, 3, and five years among patients with colorectal cancer, Monastir (2002-2014; 2022).

	≥ 1 year: % (95% CI)	≥ 3 years: % (95% CI)	≥ 5 years: % (95% CI)
All (N = 448)	64 (62.3 −66.2)	48.3 (46.3 −50.0)	21.1 (19.3-22.9)
Age groups			
< 40	83.3 (79.5–87.1)	74.0 (69.5–78.5)	59.7 (53.8 −65.6)
40-64	63.3 (60.4–66.2)	48.0 (44.9–51.0)	28.1 (25.1–31.2)
≥ 65	57.1 (54.0–60.2)*	39.0 (35.9–42.1)*	4.9 (3.5–6.3)*
Gender			
Male	65.1 (62.5-67.7)	48.2 (45.5-50.9)	22.2 (19.6-24.8)
Female	62.4 (59.6-65.2)	48.5 (45.6 −51.4)	20.0 (17.3-22.7)
Topography			
C18.0 Cecum	66.6 (65.4-67.8)	53.3 (52.0-54.6)	24.2 (22.9 - 25.5)
C18.2 Ascending colon	61.9 (51.4-72.4)	47.6 (37.5-57.5)	16.5 (7.3–25.7)
C18.6 Descending colon	60.0 (53.0–67.0)	53.8 (23.1 −76.9)	23.1 (11.9 - 46.2)
C18.7 Sigmoid Colon	72.7 (57.6 −87.9)	50.0 (43.0-57.0)	26.3 (19.1 - 33.5)
C19.9 Rectosigmoid junction	45.0 (33.9–56.1)	40.0 (29.1–50.9)	6.0 (1.8 - 10.4)
C20.9 Rectum	62.5 (56.4- 68.6)	42.2 (36.0–48.4)	16.5 (10.3–21.8)

CI: Confidence interval; *: p<0.05. missing values for date of death (n= 174; 27.9%).

**Fig 3:**
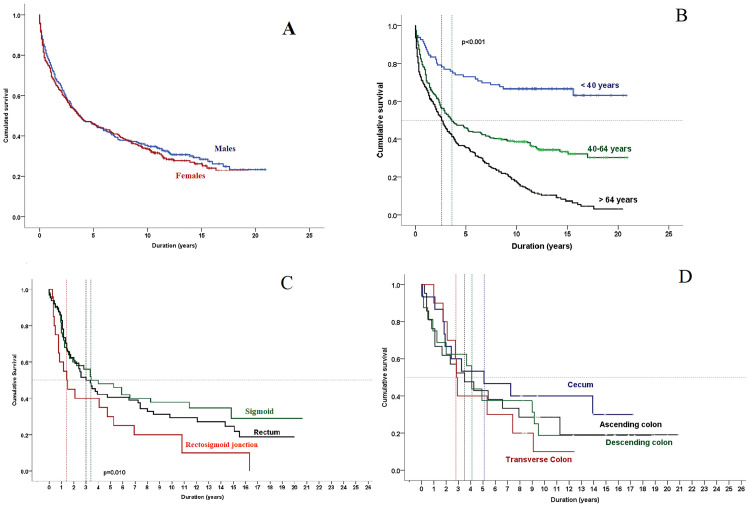
Median survival of colorectal cancer cases according to sex, ages groups and tumor localization. Kaplan–Meier survival curves for CRC patients in Monastir, 2002–2019. (A) Survival by sex, showing an equal survival among males compared with females. (B) Survival by age group, demonstrating significantly better survival among patients younger than 40 years compared with older groups (p < 0.001). (C) Survival by tumor site in the recto-sigmoid region, with the best outcomes for sigmoid cancers and poorer survival for recto-sigmoid junction tumors (p = 0.010). (D) Survival by colon subsite, indicating variability across anatomical locations, with higher survival for cancers of the cecum compared with the transverse colon.

Median survival was 3.75 years (IQR: 2.8–4.7). In subgroup analyses, survival was equal in males (3.75 years (IQR: 2.6–4.9)) and females (3.75 years (IQR: 2.3–5.1) (p = 0.510). Elderly patients had a shorter median survival of 2.6 years (IQR: 2.0–3.2) compared with 3.6 years (IQR: 2.3–4.9) in the 40–65 age group (p < 0.001). A significant difference was observed according to specific localizations (p = 0.010) (Fig 3).

## Discussion

### Main results

CRC accounted for 73.1% of digestive tract cancers. ASIR was 13.53 per 100,000 PY (95% CI: 9.8–17.20) being 14.90 in males (95% CI:11.0–18.8) and 11.81 in females (95% CI: 8.4–15.2). ASMR reached 9.61 per 100,000 (95% CI: 6.5–12.71) being 10.60 in males (95% CI: 7.3–13.86) and 8.66 in females (95% CI: 5.7–11.60). CIR increased significantly over time (APC: + 6.09 (95% CI:0.64–11.82)), especially among 40–64 years age group (APC: + 9.7% (95% CI:3.5;17.9)). Predicted ASIR is expected to reach 40.00 per 100,000 PY (95% CI: 33.7–46.3) in 2025 and 60.41 per 100,000 PY (95% CI:52.-68.2) by 2030. DALYs due to LC totaled 6609 (167.0/100,000 PY), with YLL accounting for 91.8% of this burden. The one year survival rate among all CRC patients was 64% (95% CI: 62.3–66.2), decreasing to 21.1% (95% CI: 19.3–22.9) at five years. Patients under 40 years had the highest survival rates, while those aged 65 years and older the lowest survival.

### Interpretation

In our series, colorectal cancer (CRC) represented 73.1% of digestive tract malignancies, followed by stomach cancer (21.8%), while other localizations accounted for 5.1%. The frequency of digestive tract cancers appears to vary inversely with luminal diameter, and slow intestinal transit has been recognized as a biological risk factor for colorectal carcinogenesis [17–19].

Beyond biological predispositions, modifiable risk factors such as obesity, smoking, and physical inactivity play a significant role in the growing burden of digestive tract cancers. Targeted health promotion and lifestyle interventions including nutritional education, physical activity campaigns, and anti-smoking initiatives are essential to reduce population level risk [20,21].

The ASIR of CCR in Monastir (2002–2014) was 13.53 per 100,000 PY, representing an increase compared with earlier Tunisian estimates by Misaaoui et al. (10.4 per 100,000 PY) [22]. This upward trend is consistent with the national rate in Tunisia in 2022 (18.4 per 100,000 PY) [10]. Our findings were similar to the CRC incidence reported in Algeria in 2014 [23], but remain lower than rates observed in Libya and high-income countries [24], while exceeding those reported in Morocco (9.6 per 100,000 PY, 2008–2012) [25] and in other low- and middle-income countries [26]. These differences may be explained by differences in CRC risk factors, including obesity, physical inactivity, smoking, and low fruit and vegetable intake [27]. No significant sex differences were observed, consistent with data from Libya (17.5 vs. 17.2/100,000 for males and females) [24] and Western Africa (4.5 vs. 3.8/100,000 PY) [26]. The slightly higher male incidence in our cohort may relate to greater tobacco consumption among men [28–30].

Left-sided colorectal cancers accounted for 70.9% of tumor sites, while adenocarcinoma was the predominant histopathological type, representing 96% of cases—findings consistent with those reported in the literature [13]. CRC increased significantly during our study period, consistent to literature [28]. This rising trend may be linked to the increasing adoption of a Westernized lifestyle [31], the growing prevalence of obesity and sedentary behavior [32,33] and higher smoking rates [34]. In Tunisia, obesity prevalence was 28.1%, while smoking affected 20.1% of adults aged ≥15 years in 2022 [35]. The sharpest increase was observed among the elderly, likely explained by longer life expectancy and the rising proportion of older adults in Tunisia [14].

The projected rise in CRC incidence, with ASIR expected to reach 40.0 per 100,000 PY in 2025 and 60.4 per 100,000 PY by 2030, The increasing trends and future projections observed in our analysis are in line with literature underscoring urgent need for health system preparedness [36,37]. Strengthening technical platforms and ensuring equitable access to screening, diagnostic and treatment facilities across regions will be critical for effective case management and cost containment. Expanding organized screening programs, particularly through the availability of FOBT, will be essential to improve early detection and better prognosis [38]. In parallel, effective primary prevention strategies including obesity prevention and the promotion of physical activity among adults [39] must be prioritized to curb the future burden of CRC.

The burden of CRC reached 6,609 DALYs (96.0/100,000 inhabitants), with 91.8% attributable to years of life lost (YLLs). The greatest impact was observed among individuals aged 40–64 years, accounting for 60% of the total (3,966 DALYs). These findings underscore the substantial indirect costs of CRC, including treatment burden after a cancer diagnosis [40] and the loss of productive years of life [41]. The economic burden is considerable at both the family and societal levels. In increasingly smaller households, the loss of a family member not only generates profound emotional distress and social isolation but also exacerbates economic vulnerability [42]. At the national level, the premature death of trained and productive individuals represents a significant loss of human capital, further amplifying the economic impact of CRC.

Our study demonstrated that overall survival remains poor. The one-year survival rate was 64% (95% CI: 62.3–66.2), and 21.1% (95% CI: 19.3–22.9) at five-years with a median survival of 3.75 years (IQR: 2.8–4.7). When compared with international data, our results highlight a substantial survival gap. A meta-analysis of 29 studies from the Eastern Mediterranean region reported a pooled five years survival of 57.3% (95% CI: 50.4–64.1) with the highest rates observed in Lebanon and Oman and the lowest in Libya [43–46]. In contrast, the U.S. National Cancer Institute reported a five years survival of 65%, reaching 91.1% for localized stages but only 15% in metastatic disease [47]. Such disparities are well documented and largely influenced by the stage at diagnosis [44]. Our findings underscore the influence of age on CRC survival, consistent with observations by Erik Frostberg. Independently of disease stage, younger CRC patients exhibited better survival outcomes compared with elderly patients [48]. The five-year survival rate observed in our cohort (21.1%) is therefore markedly lower than figures reported in neighboring and high-income countries, where survival typically ranges between 50% and 65% for all stages combined [49,50]. Several factors may explain this gap. First, delayed diagnosis is frequent in Tunisia, where most patients present with locally advanced or metastatic disease at first consultation [51]. These late presentations are often linked to low awareness of colorectal symptoms, sociocultural barriers, and insufficient coverage of early detection services [52]. Second, access barriers including limited endoscopic capacity, delays in pathology reporting, and uneven distribution of oncology and surgical services can postpone diagnosis and initiation of curative treatment. Third, therapeutic delays, due to long waiting times for surgery or chemotherapy, may further compromise outcomes [53]. These findings underscore the urgent need to strengthen early detection and improve timely access to diagnosis and treatment across the cancer care continuum.

Since 2016, Tunisia has introduced a national colorectal cancer (CRC) screening program based on fecal occult blood testing (FOBT) for individuals aged 50–74 years, with colonoscopy recommended for positive cases [51] Although screening programs have consistently proven effective in reducing premature mortality—for instance, U.S. data showed a 52.4% reduction in CRC mortality between 2000 and 2015 following the expansion of screening coverage [54] the Tunisian program does not overlap with our data collection period, and therefore, its impact cannot yet be assessed. To enable meaningful evaluation in the coming years, the program must be strengthened, as it remains in its early phase with limited population coverage and incomplete integration into primary care. In the pilot phase conducted in the Tunis region, only 23% of eligible individuals participated [27]. Reported barriers included low public awareness, refusal of colonoscopy after a positive FOBT, long waiting times, and the lack of availability of colonoscopy without or under anesthesia in the public sector. These implementation challenges have likely limited the program’s current impact on mortality reduction. For successful colorectal cancer screening programs [55], strengthening early detection should be a national priority. Integrating screening into preventive medicine consultations would improve feasibility and coverage. Enhanced endoscopy capacity, pathology infrastructure, and systematic follow-up are essential for timely diagnosis and management. Leveraging an electronic national cancer registry with standardized data collection and trend analysis will enable effective monitoring and resource allocation. Coupled with public awareness campaigns and professional training, these measures could substantially improve the Tunisian screening program and align CCR outcomes with international benchmarks.

### Strengths and limitations

To our knowledge, this is the first population-based investigation in Monastir to provide an in-depth evaluation of the CRC burden over two decades (2002–2022). By combining exhaustive incidence data (2002–2014) with extended follow-up of vital status until 2022, the study offers reliable external validity. Its longitudinal design allowed detailed assessment of incidence, mortality, and survival trends through internationally recognized indicators (ASIR, ASMR, APC), ensuring comparability with international research. Projections up to 2025 and 2030 further strengthen its utility for health policy and planning. The addition of survival analysis brings essential information on CRC prognosis. Despite these strengths, some limitations should be acknowledged. Notably, 27.9% of cases had missing values for the date of death, which may have introduced a potential selection bias. The absence of staging CRC data restricts interpretation of survival patterns, and the lack of information on individual risk factors prevents causal analysis. Finally, because the study is region-specific, the extrapolation of results to other populations should be made with caution.

## Conclusion

CRC is the leading digestive tract malignancy, with rising incidence and poor survival compared to global levels. Projections indicated a dramatic increase by 2030. The heavy burden in terms of DALYs and premature mortality highlights the urgent need to strengthen prevention and control strategies. Expanding organized CRC screening programs, ensuring equitable access to colonoscopy (including sedation), and addressing barriers to participation are critical to improving early detection and prognosis. In parallel, primary prevention through obesity reduction, smoking cessation, and promotion of physical activity should be prioritized to curb the rising trend.

## Supporting information

S1 Table Goodness-of-fit statistics for Poisson log-linear regression models (Age-Period-Cohort projections). (DOCX)

S1 Data Dataset of colorectal cancer cases including demographics, ICD-10 classification, tumor topography, morphology, and survival status. (XLSX)

S1 Appendix Data access and merging process. (TIF)

## Abbreviations

CRC: Colorectal Cancer
HICs: high-income countries
LMICs: low- and middle-income countries
FIT: fecal immunochemical test
ASIR/ASMR: Age-Standardized Incidence Rate/ Age-Standardized Mortality Rate
CIR/ CMR: Crude Incidence Rate/ Crude Mortality Rate
APC: Annual Percent Change
DALYs: Disability-Adjusted Life Years
YLL/YLD: Years of Life Lost/Years Lived with Disability
95% CI: 95% Confidence Interval
IQR: Interquartile Range
