# Supplementary material for: Colorectal cancer incidence, survival analysis and predictions (Monastir, Tunisia: 2002–2030)
Source: PLoS One. 2026 Jan 13;21(1):e0339603. doi: 10.1371/journal.pone.0339603 (PMC12798969; doi:10.1371/journal.pone.0339603)
Supplement: S1 Table — (DOCX) [file pone.0339603.s001.docx]

Across all subgroups, the Poisson log-linear regression models demonstrated good fit, as reflected by low AIC and BIC values and acceptable deviance and Pearson χ² statistics. These results confirm that the models adequately captured temporal variations in incidence without evidence of over-dispersion or poor calibration.

**Table 1. Goodness-of-fit statistics for Poisson log-linear regression models (Age-Period-Cohort projections)**

| **Subgroup** | **Deviance (df = 11)** | **Pearson χ² (df = 11)** | **Log-likelihood** | **AIC** | **AICC** | **BIC** | **CAIC** |
| --- | --- | --- | --- | --- | --- | --- | --- |
| **All cases** | 70.909 | 67.948 | -74.390 | 152.781 | 153.981 | 153.911 | 155.911 |
| **Males** | 73.629 | 60.920 | -71.213 | 146.425 | 147.625 | 147.555 | 149.555 |
| **Females** | 39.225 | 43.525 | -53.684 | 111.369 | 112.569 | 112.499 | 114.499 |
| **< 40 years** | 42.769 | 41.080 | -48.073 | 100.145 | 101.345 | 101.275 | 103.275 |
| **40–64 years** | 42.769 | 41.080 | -48.073 | 100.145 | 101.345 | 101.275 | 103.275 |
| **≥ 65 years** | 17.557 | 16.369 | -40.944 | 85.888 | 87.088 | 87.018 | 89.018 |

AIC: Akaike Information Criterion; AICC: small-sample corrected AIC; BIC: Bayesian Information Criterion; CAIC: Consistent AIC.
