# Supplementary figures and images for: Colorectal cancer incidence, survival analysis and predictions (Monastir, Tunisia: 2002–2030)
Source: PLoS One. 2026 Jan 13;21(1):e0339603. doi: 10.1371/journal.pone.0339603 (PMC12798969; doi:10.1371/journal.pone.0339603)

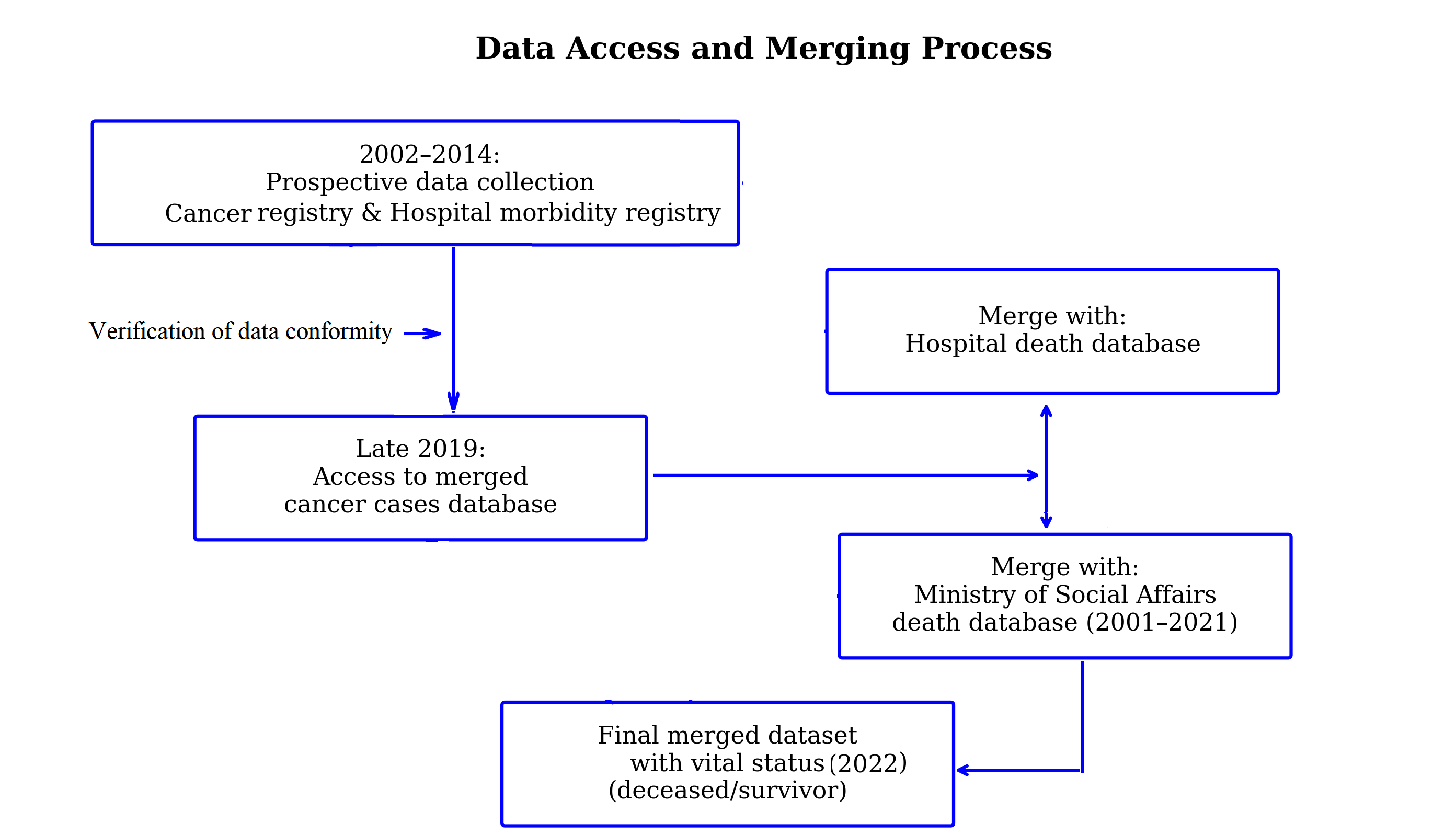

Supplement: S1 Appendix — (TIF) [file pone.0339603.s003.tif]
